# Examining the impact of specific types of item-writing flaws on student performance and psychometric properties of the multiple choice question

**DOI:** 10.15694/mep.2018.0000225.1

**Published:** 2018-10-02

**Authors:** Hannah Pham, James Besanko, Peter Devitt

**Affiliations:** 1 University of Adelaide; 2 Royal Adelaide Hospital; 3 Royal Adelaide Hospital

**Keywords:** Multiple choice question, Item writing flaws, Medical assessment, Psychometrics, Item difficulty, Item discrimination

## Abstract

This article was migrated. The article was marked as recommended.

**Background**: Item-writing flaws (IWFs) are common in multiple choice questions (MCQs) despite item-writing guidelines. Previous studies have shown that IWFs impact validity as observed through student performance, item difficulty, and discrimination. Most previous studies have examined IWFs collectively and have shown that they have a diverse impact. The aim of the study was to determine if the effects of individual types of IWFs are systematic and predictable.

**Method**: A cross-over study design was used. 100 pairs of MCQ items (with and without an IWF) were constructed to test 10 types of IWFs. Medical students were invited to participate in a mock examination. Paper A consisted of 50 flawed followed by 50 unflawed items. Paper B consisted of 50 unflawed followed by 50 flawed items. The effect of each of the IWFs on mean item scores, item difficulty and discrimination were examined.

**Results**: The hypothesised effect of IWFs on mean item scores was confirmed in only 4 out of 10 cases. ‘ *Longest choice is correct*’, *‘Clues to the right answer (Eponymous terms)’* and ‘ *Implausible distractors*’ positively impacted, while *‘Central idea in choices rather than stem’* negatively impacted mean item scores. Other flaws had either the opposite or no statistically significant effect. IWFs did not impact item difficulty or discrimination.

**Conclusion**: The effect of IWFs is neither systematic nor predictable. Unpredictability in assessment produces error and thus loss of validity. Therefore, IWFs should be avoided. Faculties should be encouraged to invest in item-writing workshops in order to improve MCQs. However, the cost of doing so should be carefully weighed against the benefits of developing programmes of assessment.

## Introduction

Multiple choice questions (MCQs) are a common question type in medical assessment. They are familiar to educators, amenable to construction and administration by computer-based technology. They enable testing of broad content and, if well-constructed, can test more than simple facts. ( Schuwirth and van der Vleuten 2003) However, a frequently cited shortcoming of MCQs concerns the phenomenon of “cueing”, which is the ability of a student to correctly recognise the answer from a list of options rather than spontaneously generate it. ( Schuwirth, van der Vleuten and Donkers 1996)

MCQs are also susceptible to errors that may occur via item-writing flaws (IWFs), which are defined as violations of standard item-writing guidelines. There are several types of item-writing guidelines, pertaining to content, formatting, style, writing the stem, and writing the choices. ( Haladyna, Downing and Rodriguez 2002) Some types of IWFs cue the student to the correct answer, which may lead to false-positive responses by favouring students who are ‘test wise’. ‘Test-wiseness’ refers to a student’s ability to recognise the answer in an MCQ without utilising their content-specific cognitive skill or knowledge. Theoretically, other types of IWFs may mislead and penalise the student. This may over or under-estimate student performance, thus introducing a source of error that compromises the validity of student scores. ( Downing 2005, Tarrant and Ware 2008)

Published literature has shown that IWFs may impact parameters including student performance, item difficulty and discrimination indices. The relationship between these measures appears to be mixed.

One study showed that although IWFs had relatively little effect on psychometric indices, up to 10-15% of students failing the MCQ examination would have passed if they were removed. ( Downing 2005) Another study analysed IWFs and student performance within subgroups. Although there were no significant statistical differences regarding difficulty of items, high-achieving students were negatively impacted by flawed tests compared to low-achievers, who were not. ( Tarrant and Ware 2008)

Most empiric evidence for single flaws exists for negative phrasing, ‘none of the above’ and ‘all of the above’. ( Haladyna, Downing and Rodriguez 2002) These were largely studied using retrospective analyses of examination items. One study showed that the use of the negative form in items testing higher order cognitive skills increased question difficulty, as it is more cognitively taxing than the corresponding positive form of the question. ( Tamir 1993) The use of the ‘none of the above option’ resulted in a decrease in difficulty and discrimination indices. ( Rich and Johanson 1990) On the other hand, students who recognised two or more options as being correct were cued to select ‘all of the above’, resulting in elevated scores, thus reducing reliability and concurrent validity of examinee scores. ( Harasym et al 1998) Another study reported heterogenous findings, with ‘writing the stem’ and ‘writing the choices’ categories having a negative impact (higher difficulty, lower discrimination) compared to other categories (content, formatting, and style concerns) which did not have an impact. ( Pais et al 2016)

The current findings in literature are diverse - they depend on the approach to examining IWFs collectively, as categories, or as single flaws, and are further confounded by differences in item content and cognitive skill level. There has not been a prospective empiric study directly examining the effects of multiple individual types of IWFs, with the aim of ascertaining if the effects of IWFs are inherent or due to methodological differences.

Whilst there is wide acceptance of the principles of effective item-writing, the frequency of poor quality items in medical assessment remains high. ( Tarrant, Knierim, Hayes and Ware 2006) Given the increasingly popular use of the MCQ format in high-stakes assessments, attention should be given to potential sources of error that can introduce construct-irrelevant variance.

The aim of this study is to provide better methodologically-grounded evidence that individual types of IWFs have a predictable and systematic effect on student performance and psychometric indices.

An understanding of the impact of individual flaws may direct item-writers in avoiding those which can have a critical impact on student performance. This may also lead to the revision of item-writing guidelines, perhaps by organisation of flaws by their impact (positive, negative, or no effect on student performance). Finally, understanding the impact will inform the content of item-writing workshops, as well as contribute to the overall cost-benefit evaluations of these exercises.

## Methods

### Participants

Year IV and Year V students in the Bachelor of Medicine, Bachelor of Surgery (MBBS) course at The University of Adelaide were invited to participate in a mock examination in October 2017.

### Sampling and recruitment

Social media was used to advertise the study, with recruitment sought on the day of the examination. Participants indicated their consent to participate on registering for the examination.

### Materials

This prospective study was conducted as a mock examination. A cross-over study design was used.

For this, 100 pairs of MCQs (200 items in total) were constructed. The content was constructed by clinicians (authors HP and JB), and then reviewed by a senior clinician (author PD) who has over 25 years’ experience in MCQ writing. The content of questions included general medicine and general surgery and was targeted at end-of-year 4 summative level. As the University of Adelaide end-of-year 5 summative level assessments encompass material from Year 4, it was felt appropriate that both cohorts were assessed on these specific content areas. Each MCQ consisted of five answer options in a single-best-answer format and was worth one mark.

Items within each pair (with and without an IWF) were matched in content. Ten pairs of items were used to test each flaw, with 10 flaws in total examined in this study. These flaws were selected from only the ‘Writing the Stem’ and ‘Writing the Choices’ categories, as these were noted by an earlier study to have a negative impact (higher difficulty, lower discrimination). ( Pais et al 2016)

Ten individual hypotheses were made about the effect of each type of IWF on student performance at the mean item levels. A ‘positive’ impact was defined as one with a higher mean score on a flawed compared to unflawed item. A ‘negative’ impact was defined as one with a lower mean score on a flawed compared to unflawed item. It was further hypothesised that IWFs which negatively or positively impact on student performance would increase or decrease difficulty, respectively, and lower discriminatory ability.

Two examination papers were constructed. Paper A consisted of 100 items, the first 50 which were flawed (covering IWFs 1-5) and the latter 50 which were unflawed items (unflawed versions of IWFs 6-10). Paper B contained 100 items, the first 50 which were unflawed (unflawed versions of IWFs 1-5) and the latter 50 which were flawed items (covering IWFs 6-10). Items 1-50 in Paper A corresponded in content to items 1-50 in Paper B. Items 51-100 in Paper A corresponded in content to items 51-100 in Paper B. The layout of the mock examinations and hypothesised effect of each IWF is shown in Table 1. A sample of a matched pair of items is shown in Table 2.

The time limit was 120 minutes for the examination.

**Table 1.** Hypothesised effect of item-writing flaws and layout of the examinations

	Type of item-writing flaw	Paper A	Paper B	Hypothesised effect on:		
				Student performance	Difficulty	Discrimination
**1**	Window dressing/Excessive verbiage	Flawed	Unflawed	Negative	Increase	Decrease
**2**	Longest choice is correct	Flawed	Unflawed	Positive	Decrease	Decrease
**3**	Clues to the right answer(pairs of options)	Flawed	Unflawed	Positive	Decrease	Decrease
**4**	None/all of the above	Flawed	Unflawed	Negative	Increase	Decrease
**5**	Negatively phrased question	Flawed	Unflawed	Negative	Increase	Decrease
**6**	Lack of direction in stem	Unflawed	Flawed	Negative	Increase	Decrease
**7**	Central idea in choices rather than stem	Unflawed	Flawed	Negative	Increase	Decrease
**8**	Out of logical/numerical order	Unflawed	Flawed	No effect	No effect	No effect
**9**	Clues to the right answer(eponymous names)	Unflawed	Flawed	Positive	Decrease	Decrease
**10**	Implausible distractors	Unflawed	Flawed	Positive	Decrease	Decrease

**Table 2.** Sample of a matched pair of items

**Item-writing flaw**	***IWF3: Clues to the right answer (pairs of options)*** *Single pair of options (two options are perceived as a pair by candidates, who limit their guess to the pair)*
**Flawed**	Which one of the following is the best way of confirming correct placement of the endotracheal tube? A. Fogging on plastic tubing B. Visualisation of symmetrical chest wall rise and fall C. Expired concentrations of oxygen D. Expired concentrations of carbon dioxide* E. Equal air entry on chest auscultation
**Unflawed**	Which one of the following is the best way of confirming correct placement of the endotracheal tube? A. Fogging on plastic tubing B. Visualisation of symmetrical chest wall rise and fall C. Direct visualisation between the vocal cords D. Expired concentrations of carbon dioxide* E. Equal air entry on chest auscultation

### Data collection procedure

#### Test sessions

The study was in the form of a mock examination, administered on an online platform at a designated time on one day in October 2017. Students were advised to enrol themselves into either group A or B based on their surname (A-M or N-Z, respectively). Students sat the examination on personal electronic devices.

#### Examination marking

The examinations were marked electronically using a simple algorithm that compared student responses with correct responses.

#### Statistics

The data was de-identified prior to data analysis.

Despite the randomisation procedure, comprehensive analyses were performed to ensure comparability of groups A and B. For this, various t-tests were performed with the intention of showing no difference regardless of how the groups and flaws were analysed.

First, paired-samples t-tests were performed between the flawed and unflawed groups of items in both Group A and B examinations (T1). Subsequently, independent samples t-tests were performed between the first half of the examination (same content but flawed in group A and unflawed in group B) and repeated this for the second half (unflawed in group A and flawed in group B) (T2) and on the total score on all items in groups A and B (Total). Finally, the first half of the examination of group A was compared with the second half of the examination of group B, and repeated for the second half of the examination of group A and first half of the examination of group B. For this, t-tests were not possible as it concerned different groups and different items (but the similarity being that they were all flawed items in the one comparison and unflawed in the other). Therefore, standard errors around the mean scores in all for groups were calculated and used to construct 95% and 68% confidence intervals (T3). In summary, the comparisons made were:

- T1: compared across different content and whether the items were flawed or not but within students of the same group
- T2: compared across different groups and whether the items were flawed or not but between students of different groups
- T3: compared across different content and different groups but with the condition flawed or unflawed.
- Total: compared across different groups and different flaws but with the same content.

As both groups had been exposed to half of the items being flawed and half being unflawed, a significant difference at the total score level was not expected. Therefore, the differences between the individual IWFs was examined. This results in 10 t-tests and would normally require either an analysis of variance or at least a Bonferroni correction. However, we were not interested in individual conclusions but rather to explore how many of our theoretical assumptions would be confirmed or not. Therefore, each comparison was entered into a straightforward sign test to test the hypothesis that effects of item writing flaws are predictable. Ten t-tests were performed to compare individual flaws eg, *IWF1* in the flawed and unflawed versions, *IWF2* in the flawed and unflawed versions, etc.

Finally, difficulty index was calculated for each item using the proportion of all students who sat each item who scored correctly, and discrimination index was calculated from point-biserial correlations, correlating the continuous total score with the binary response (correct or incorrect).

## Results/Analysis

### Participation

263 Years IV and V medical students participated in this mock examination. 106 sat Paper A and 157 sat Paper B.

### Items

Two items were removed from the *IWF5* group of questions due to transcription errors. A total of 98 pairs of items were analysed.

### Descriptive statistics

Descriptive statistics are provided in Table 3.

**Table 3.** Descriptive statistics

Group	Items tested	n	Minimum	Maximum	Mean	Standard deviation	Cronbach’s alpha
A	IWFs 1-5 (Flawed)	106	31.25	81.25	57.49	10.14	0.638
A	IWFs 6-10 (Unflawed)	106	28.00	78.00	56.72	10.30	0.643
B	IWFs 1-5 (Unflawed)	157	25.00	77.08	55.27	10.52	0.660
B	IWFs 6-10 (Flawed)	157	16.00	80.00	55.80	10.06	0.632

### Comparability of Groups A and B

**Figure 1.**
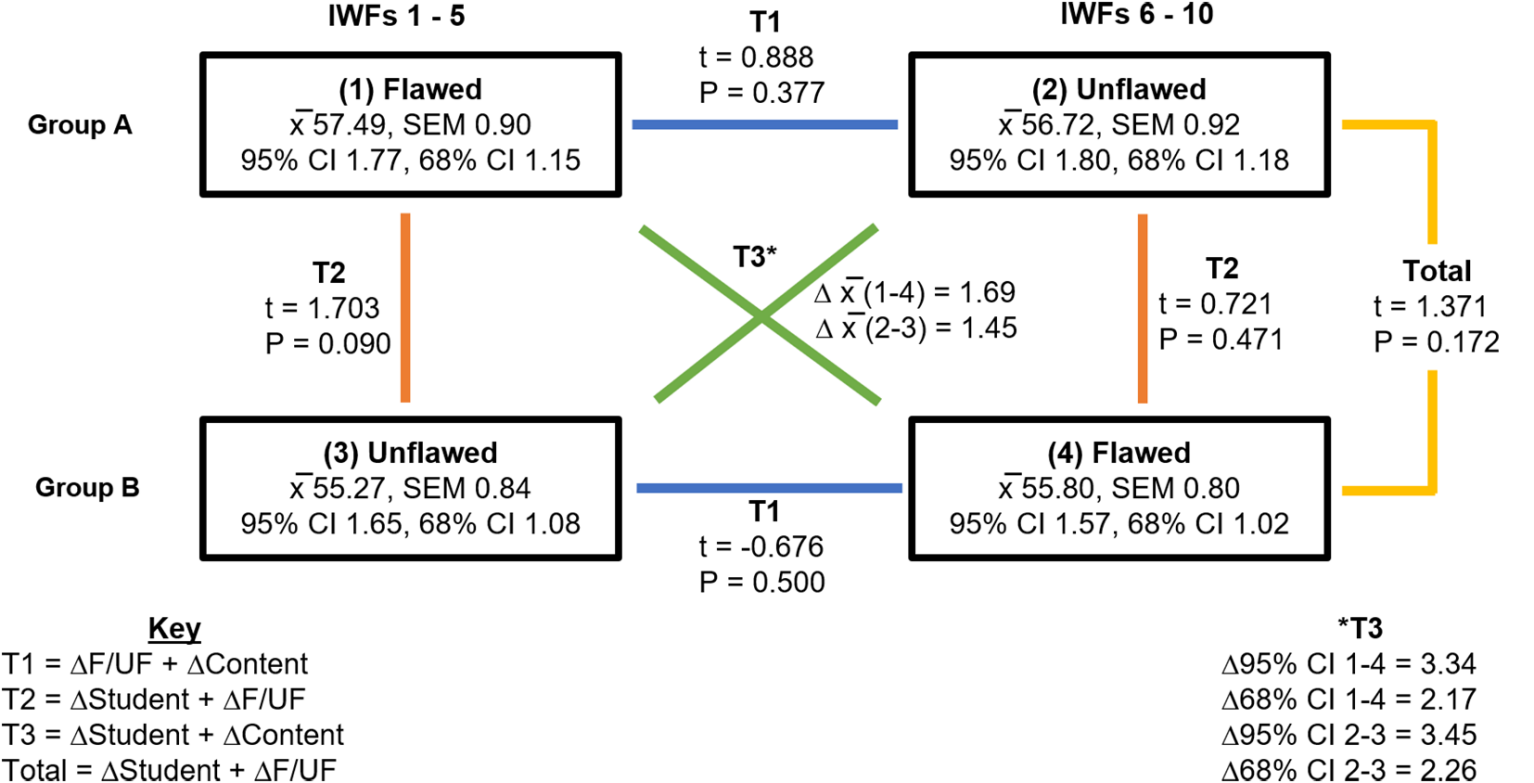
Various t-tests within and across Groups A and B examinations

Figure 1 demonstrates the various t-tests performed to assess comparability of groups A and B. Comparisons between the flawed and unflawed items in both groups (T1), between the flawed and unflawed versions of items across groups (T2), and between total scores in each examination (Total) showed non-significance. At the levels of the 95% and 68% confidence intervals, there were no differences when comparing flawed groups of items and unflawed groups of items (T3). As all the differences were not significant, we concluded that the groups were comparable and that randomisation had been successful.

### Impact of specific item-writing flaws on student performance at the level of mean item performance

The impact of 10 specific IWFs on student performance at the mean item level is summarised in Table 4 (reported in column ‘actual effect’). Statistically significant differences were found in five cases. In four of these cases, the effect of the flaw was in the anticipated direction (positive, negative, or nil effect). ‘ *IWF2: Longest choice is correct*’, *‘IWF9: Clues to the right answer (Eponymous terms)’* and ‘ *IWF10:Implausible distractors*’ positively impacted, while *‘IWF7: Central idea in choices rather than stem’* negatively impacted mean item scores. ‘ *IWF5: Negatively phrased question*’ positively impacted mean item scores, contrary to the hypothesis.

**Table 4.** t-tests for each item-writing flaw

IWF	t score	Sig	Item mean (flawed)	Item mean (unflawed)	Hypothesised effect	Actual effect
**1**	1.919	.056	0.6132	0.5745	Negative	Nil
**2**	1.997	. **047**	0.6623	0.6185	**Positive**	**Positive**
**3**	-1.798	.073	0.5038	0.5395	Positive	Nil
**4**	-1.389	.166	0.4943	0.5178	Negative	Nil
**5**	4.684	. **000**	0.6073	0.5032	**Negative**	**Positive**
**6**	1.292	.198	0.5815	0.6094	Negative	Nil
**7**	7.013	. **000**	0.5083	0.6396	**Negative**	**Negative**
**8**	.713	.477	0.4720	0.4868	Nil	Nil
**9**	-3.636	. **000**	0.7134	0.6302	**Positive**	**Positive**
**10**	-2.236	. **026**	0.5146	0.4698	**Positive**	**Positive**

### Impact of item-writing flaws on difficulty and discrimination

**Figure 2.**
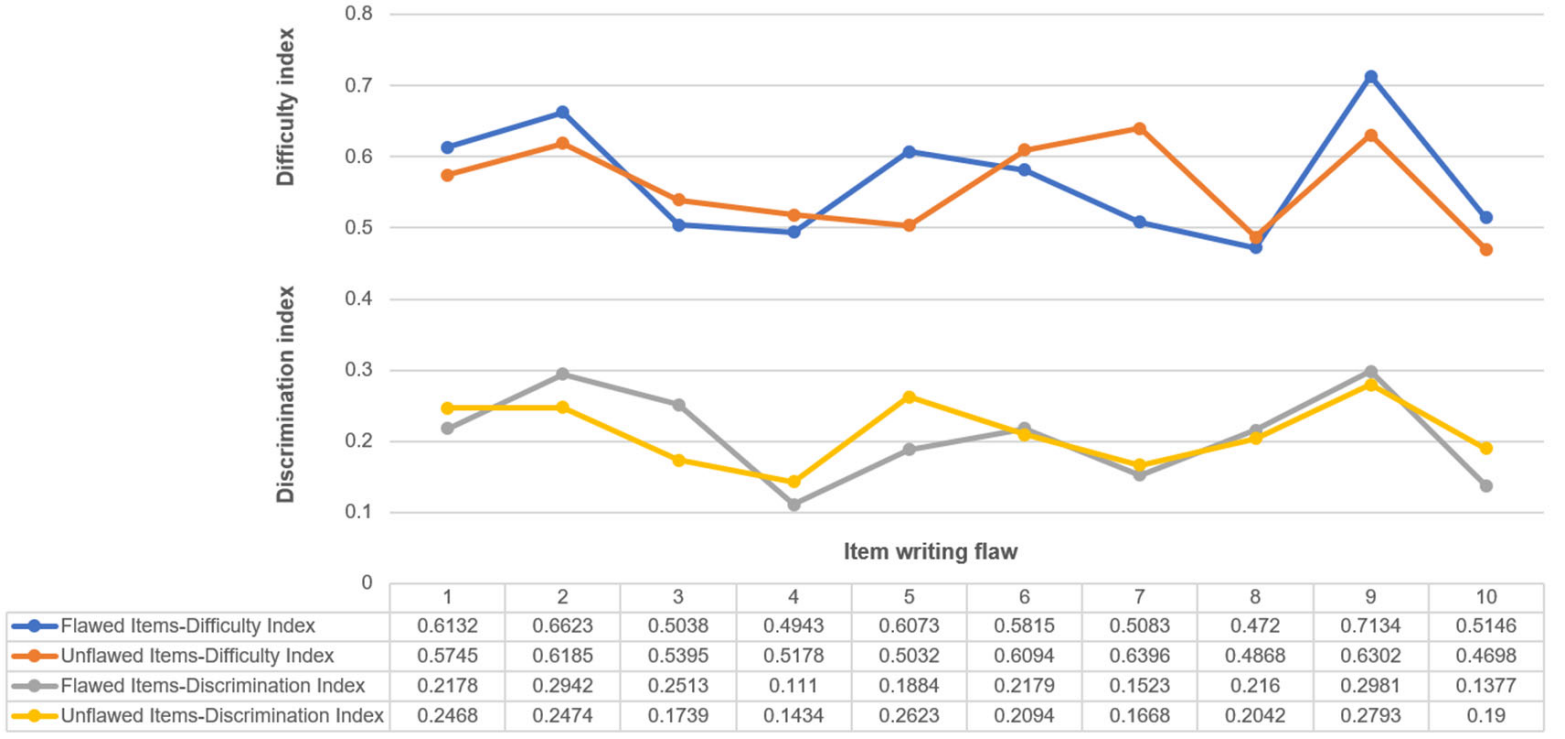
Difficulty and discrimination indices by type of item writing flaw

There were no statistically significant differences in difficulty or discrimination indices when comparing flawed to unflawed groups of items, as shown in Table 5, Table 6, and Figure 2.

**Table 5.** Differences of marginal means of difficulty indices across item-writing flaws

IWF	Difficulty index (Flawed)	Difficulty index (Unflawed)	Difference in difficulty indices	Standard Error	Lower 95% confidence level	Upper 95% confidence level	P value
**1**	0.6132	0.5745	0.03869	0.1020	-0.1612	0.2386	0.7044
**2**	0.6623	0.6185	0.04379	0.1020	-0.1561	0.2437	0.6676
**3**	0.5038	0.5395	-0.03572	0.1020	-0.2356	0.1642	0.7262
**4**	0.4943	0.5178	-0.02349	0.1020	-0.2234	0.1764	0.8178
**5**	0.6073	0.5032	0.1041	0.1140	-0.1194	0.3276	0.3611
**6**	0.5815	0.6094	-0.02791	0.1020	-0.2278	0.1720	0.7844
**7**	0.5083	0.6396	-0.1313	0.1020	-0.3312	0.0684	0.1978
**8**	0.4720	0.4868	-0.01482	0.1020	-0.2147	0.1851	0.8845
**9**	0.7134	0.6302	0.08319	0.1020	-0.1167	0.2831	0.4147
**10**	0.5146	0.4698	0.04484	0.1020	-0.1550	0.2447	0.6602

**Table 6.** Differences of marginal means of discrimination indices across item-writing flaws

IWF	Discrimination index (Flawed)	Discrimination index (Unflawed)	Difference in discrimination indices	Standard Error	Lower 95% confidence level	Upper 95% confidence level	P value
**1**	0.2178	0.2468	-0.02898	0.05189	-0.1307	0.07273	0.5765
**2**	0.2942	0.2474	0.04684	0.05189	-0.05487	0.1485	0.3667
**3**	0.2513	0.1739	0.07738	0.05189	-0.02433	0.1791	0.1359
**4**	0.1110	0.1434	-0.03242	0.05189	-0.1341	0.06929	0.5321
**5**	0.1884	0.2623	-0.07388	0.05802	-0.1876	0.03983	0.2029
**6**	0.2179	0.2094	0.008459	0.05189	-0.09325	0.1102	0.8705
**7**	0.1523	0.1668	-0.01451	0.05189	-0.1162	0.08720	0.7798
**8**	0.2160	0.2042	0.01180	0.05189	-0.08991	0.1135	0.8201
**9**	0.2981	0.2793	0.01877	0.05189	-0.08294	0.1205	0.7176
**10**	0.1377	0.1900	-0.05230	0.05189	-0.1540	0.04940	0.3135

## Discussion

In our study we made 10 predictions on the anticipated direction of effect of specific types of IWFs on student performance with the intent to see how often these theoretical predictions would be present in the data. The predictions were confirmed at the mean item level in only four out of 10 cases. In six out of 10 cases, the anticipated effect was not shown. IWFs made no statistically significant difference to difficulty or discrimination indices. This study has shown that a solid prediction of the effect of single types of IWFs on student performance, difficulty, and discrimination is difficult to achieve. At the level at which this study was performed, the effect is heterogenous. From an overall perspective, it is safe to conclude that IWFs do not have a systematic nor a predictable effect on student performance. This is a reason to avoid any IWFs as unpredictability in assessment is a threat to validity.

Here, we examine some of the flaws in closer detail.

When examining *IWF2: Longest choice is correct*, our results support the prevailing hypothesis that this flaw has a positive impact on student performance. It was hypothesised that item-writers might include additional information in the key to distinguish it from a similar (perhaps partly correct) distractor. Our findings support ongoing adherence to the guideline of making the choices in an item of equal length, which is consistent with Haladyna, Downing, and Rodriguez’s (2002) review of this guideline’s validity evidence.

A previous study showed that the use of the negative form in items testing higher order cognitive skills increased question difficulty, as it is more cognitively taxing than the corresponding positive form of the question. ( Tamir 1993) Our study that use of *IWF5: Negatively phrased question* resulted in an increase in the mean item score. One study noted that item-writers ‘often make the correct answer (the incorrect option in a negatively phrased question) so obviously incorrect that students can easily spot the answer and the question becomes too easy to adequately discriminate between the most and least able students in the test’. ( Tarrant, Knierim, Hayes and Ware 2006) This may explain why the use of negatively phrased questions in this study had a positive effect on student performance.

*IWF7: Central idea in choic*es *rather than stem* negatively impacted mean item scores between flawed and unflawed groups. When the central idea is in the choices rather than the stem, the student may have to reason through five possibly unrelated statements - which resembles a multiple true/false-type of MCQ. The testing objectives of the item increase multi-fold and so too does the cognitive load required to answer the question, in contrast to a single-best-answer format where the stem would likely be posed as a clinical scenario and the question asked is isolated to one objective. The adverse features of the true/false MCQ that make it inappropriate in many assessment settings include inadvertent assessment of trivial knowledge and poor discrimination of students. ( Chandratilake, Davis and Ponnamperuma 2011) Our findings support the ongoing adherence to the guideline that the central idea of the item should be in the stem rather than choices.

*‘Avoid giving clues to the right answer’* is a broad item-writing guideline with six variations noted in a validated taxonomy of item-writing guidelines. ( Haladyna, Downing, and Rodriguez 2002) We identified the use of eponymous terms as a potential violation of this guideline. Our study has shown that even within a single type of guideline, the effect is heterogenous. While *IWF3: Clues to the right answer (Pairs of options)* did not impact mean item scores, *IWF9: Clues to the right answer (Eponymous terms)* had a positive impact, which supports our hypothesis. The hypothesis was made on the basis that students would be cued to the correct answer. Proponents for the use of eponymns in practice argue that it can make unmemorable concepts memorable, label complex concepts, and promote an interest in medical history. ( Thomas 2016) Eponyms are used frequently in daily patient care and scientific literature. However, they can be unreliable in case of information transfer. At the level of item-writing, students who are not familiar with the original meaning of the eponym may be adversely affected in their performance on the item. One study examined whether the original meaning of eponymous terms had been preserved in contemporary literature and found that the use of such terms is inconsistent, and the understanding of its meaning and content varying between surgeons. ( Somford et al 2016) Given the potentially diverse effect of eponymous terms, where possible, they should be avoided in item-writing.

The presence of *IWF10: Implausible distractors* had a positive impact on mean scores between the flawed and unflawed groups of questions, which is consistent with our hypothesis. However, it did not impact item difficulty or discrimination. This contrasts with Rush, Rankin and White’s (2016) finding that implausible distractors decreased the probability of writing discriminating questions. Determining plausibility relies on the application of scientific knowledge to a new hypothesis to guide us in how likely it is to be true. The student who has no content knowledge of the item but who can effectively ‘rule out’ options based on their assessment of plausibility can then narrow down their guessing to fewer options than the five which are frequently employed in MCQs. Haladyna, Downing, and Rodriguez (2002) explored the evidence for the most desirable number of options in an MCQ - an area that has received a significant amount of attention in medical education literature. They conclude that three options is sufficient in most instances, and that the effort of developing the fourth option is ‘probably not worth it’. They state that ‘it is very unlikely that item writers can write three distractors that have item response patterns consistent with the idea of plausibility’. Therefore, the minimum required number of options in an item should be considered in the context of the ability to develop plausible distractors. Implausible distractors should be avoided as its use has been shown in this study to lead to an increase in students’ mean item scores.

One flaw that is worthy of further discussion is *IWF4: None/all of the above.* When this flaw was presented as one of four *incorrect* options (ie, as a distractor) in each five-option MCQ, there was no difference in mean item scores. The use of this option as either the distractor (incorrect answer) or key (correct answer) will likely impact on its effect on student performance. Students who select ‘none of the above’ or all of the above’ may be those who are cued and believe this to be the correct answer. If it is used as the key the flaw may therefore artificially elevate a student’s performance, but if used as a distractor, may penalise the student. One study suggested that cautious use of ‘none of the above’ could be compatible with good classroom testing when consistent with instructional objectives and when items would otherwise be too easy ( Frary 1991). Another study suggested that the use of this option be restricted to “items that can logically include one correct option only”. ( Kolstad and Kolstad 1991) In a review of validity evidence, it was concluded that ‘none of the above’ should be avoided by novice item writers, and ‘all of the above’ avoided altogether. ( Haladyna, Downing and Rodriguez 2002) The findings of this study are inconclusive regarding the use of ‘none of the above’ or ‘all of the above’.

Finally, *IWF1: Window dressing/Excessive verbiage*, *IWF6: Lack of direction in stem* and *IWF8: Out of logical/numerical order* did not have a statistically significant impact on mean item scores. Whilst these findings were unexpected considering their presence in validated item-writing guidelines, this reiterates the primary conclusion drawn from this study: that the effects of individual types of IWFs are neither systematic nor predictable.

Most examinations contain a high frequency of IWFs. One study examined four basic science examinations administered to medical students and reported a frequency of IWFs ranging from 36-65% of questions. ( Downing 2005) Another study of 2770 MCQs from tests administered to nursing students and showed that almost half (46.2%) the questions contained IWFs. ( Tarrant and Ware 2008) Most examinations will inadvertently contain many of various types of flaws so the comparison with a ‘unflawed examination’ in our analysis is artificial. Flaws may offset each other or have an additive negative or positive effect on overall student performance. Theoretically, if an assessment contained only those flaws which had a ‘negative’ impact on student performance, this may subsequently influence pass-fail outcomes. Likewise, there may be no overall effect if a composition of IWFs with both positive and negative effects are used in an examination. Although theoretically possible to predict that an *x* number of a certain composition of flaws will result in a deviation/error margin of *x* in overall student performance, it is not a feasible nor realistic undertaking - as the inclusion of IWFs in summative examinations are not typically deliberate or conscious decisions. It is common practice to review all items prior to administration in an examination and then perform key validation afterwards. This study did not evaluate whether there was any effect of individual types of IWFs on overall performance, as this would be entirely dependent on the number of flaws to total number of items - which is an artificial analysis. As this study has shown, the predictions made about anticipated effect (positive, negative, nil) were confirmed in only 4 of the 10 IWFs examined. Although there were several types of IWFs not examined in this study, it could be extrapolated from the current findings that a repeated study with 10 different IWFs would yield similarly unpredictable effects - which is not dissimilar from the current mixed findings in literature. This makes an argument for avoidance of all types of IWFs.

The placement of IWFs in their groups numbered 1-5 and 6-10 was arbitrary and not determined by their hypothesised effect. Despite each student only being exposed to half of all flaws (either IWFs 1-5 or IWFs 6-10), the various t-tests performed in our analysis confirmed that Groups A and B were comparable. An important comparison is between the mean scores of these groups: the mean score of the flawed items in Group A (IWFs 1-5) was 57.5 (out of 98) compared to 55.8 in Group B (IWFs 6-10), 56.7 and 55.3 for unflawed items in Groups A and B, respectively. The likeness of these mean scores despite significant variation in the type and presence of flaws in the examinations used in this study suggests that the mechanisms by which flaws act is truly unpredictable.

### Translating findings into practice

The MCQ format remains popular in medical assessment, particularly with repeated studies supporting its equivalence to the open-ended format in testing higher order cognitive skills. ( Hift 2014) Many faculties continue to utilise the MCQ at high-stakes examination levels. While this reflects well-established findings in literature, the use of the MCQ as a sole format is not infallible.

A combination of techniques will always be required as no single assessment method is appropriate for assessing all the skills, knowledge, and attitudes needed in medicine. ( Van der Vleuten 1996) Programmatic assessment is an “integral approach to the design of an assessment program with intent to optimise its learning function, its decision-making function and its curriculum quality-assurance function”. (Van der Vleuten et al 2015) It is built on the philosophy that the whole is more than the sum of its parts. The combination of various assessment instruments is carefully coordinated, accepting that each instrument has its own strengths, weaknesses, and purpose in a programme. This approach is not without its costs, however, as it can only be afforded if the resources for assessment are re-distributed.

The effects of IWFs are neither systematic nor predictable. Unpredictability carries with it risk of potential error that can compromise validity, and as such, the need to minimise IWFs should continue to be emphasised. There were small but measurable changes in mean scores when specific IWFs were present. Furthermore, if students can identify issues with the format, style, content, stem or options, then face validity is lost - irrespective of whether the IWF should theoretically make a difference to mean scores. Institutions should ensure that the assessments they deliver are informed by academic literacy.

This study has shown that there is an ongoing need to improve MCQs. Item-writers should be encouraged to adhere to item-writing guidelines, irrespective of MCQs being utilised as a sole format or within a programme of assessment. It has been shown that questions constructed by trained faculty are significantly higher in quality than those written by untrained faculty. ( Jozefowicz et al 2002) In fact, the utility of long term faculty development programs to improve quality of MCQs items’ writing has been shown to significantly improve difficulty index values and discrimination indices. ( Abdulghani et al 2015, Abdulghani et al 2017, Ali and Ruit 2015) Faculties should be encouraged to invest in item-writing workshops in order to improve MCQs. However, a common reality is that faculties are increasingly hard-pressed to deliver time and cost-effective workshops for clinicians, who may not be expert item-writers, and academics, who may not have clinical expertise. Therefore, with the understanding that no one test instrument is perfect, the cost of pursuing the ‘perfect’ MCQ examination should be carefully weighed against the benefits of developing programmes of assessment.

### Strengths and limitations

The strength of this study originates from its prospective empirical approach. It has examined IWFs discretely rather than categorically, and with acceptable reliability.

The generalisability of the findings is limited by participant origin from a single institution. However, the sample size is an accurate reflection of medical assessment at a tertiary level. Any larger class size would be artificial and negate ecological validity.

The study was limited by its randomisation procedure (alphabetical) which resulted in uneven numbers in each group. Participants were made aware of the nature of the study, which could have cued their behaviour in the mock examination. There was a risk that participants could recognise and respond to the nature of the items. The presentation of flawed items in succession (ie, in the first half of the examination for Group A, and in the second half for group B) in the mock examinations could also have contributed to a change in response behaviour.

The study did not evaluate the interaction between IWFs and cognitive skill level (as defined by Bloom’s taxonomy). One study found that as item complexity increased, item difficulty and discrimination values increased. ( Rush, Rankin and White 2016) An analysis of the cognitive skill level of items which have undergone removal or addition of specific types of IWFs should be undertaken, to determine if there is a predictable relationship between item complexity and specific types of IWFs. For example, it could be hypothesised that items testing higher order cognitive skills are less prone to IWFs compared to those testing lower order cognitive skills, such as knowledge/recall.

Finally, the study did not evaluate individuals’ specific content knowledge. If a student has strong content knowledge in a particular area, they may be able to compensate for an IWF, even if the IWF should theoretically hinder them. Similarly, if they are weak in a particular area, they may not be cued by an IWF that should theoretically help them. Comparisons to student performance on summative assessments and/or student self-reports of content-specific knowledge could be explored as another variable impacting performance on flawed items.

### Future research

An area for future research is the exploration of how flawed items impact on the cognitive processes elicited in a student, perhaps with a ‘think aloud’ study approach. It is not clear if there is conscious or deliberate behaviour from test-takers in either utilising item-writing flaws which cue to the correct answer or recognition (and avoidance) of the flaws which make up ‘trick questions’.

Future studies should address the interaction between the flaw, cognitive skill level of the item, individuals’ content-specific knowledge and thus performance.

## Conclusion

This study has shown that the effect on student performance and psychometric indices, including item difficulty and discrimination, of 10 specific types of IWFs is neither systematic nor predictable. As unpredictability in assessment is a threat to validity, IWFs should be avoided.

Faculties should be encouraged to invest in item-writing workshops to improve MCQs. However, the costs of doing so should be carefully weighed against the benefits of developing programmes of assessment.

## Take Home Messages

• Item-writing flaws are a threat to the validity of examinations and should be avoided as their effects on student performance, item difficulty and discrimination are neither systematic nor predictable.
• Faculties should be encouraged to adhere to item-writing guidelines.
• Item-writers should be made aware of the potential implications of item-writing flaws, and the need to avoid all types emphasised.
• Faculties should be encouraged to invest in item-writing workshops where items are reviewed, critiqued, and potential flaws identified. However, the costs of doing so should be carefully weighed against the benefits of developing programmes of assessment.

## Notes On Contributors

**Hannah Pham**, MBBS MClinEd, is a clinical lecturer at the University of Adelaide.

**James Besanko**, MBBS is an intern at the Royal Adelaide Hospital.

**Peter Devitt**, MBBS FRCS (Eng.) MS (Lond) FRACS is a general surgeon and Associate Professor at the University of Adelaide.
