# Supplementary material for: Examining the impact of specific types of item-writing flaws on student performance and psychometric properties of the multiple choice question
Source: MedEdPublish (2016). 2018 Oct 2;7:225. [Version 1] doi: 10.15694/mep.2018.0000225.1 (PMC10711986; doi:10.15694/mep.2018.0000225.1)
Supplement: Peer review history

# Reviewer report: Reviewer response for version 1

**DOI:** 10.21956/mep.19686.r29417

**Published:** 2018-10-20

**Authors:** Michael SH Wan

**Affiliations:** 1 University of Notre Dame

This review has been migrated. The reviewer awarded 4 stars out of 5 A well written study of a commonly used format of assessment in medical schools - MCQ.Known common item flaws were discussed in detail and by reading the study will remind many on MCQ questions development and review.The randomised cross over study (although only in one school and with a relatively small number of items) was well designed.I agree with previous reviewers that item banks have improved over the years with more efforts being put in to improve quality of items.

Reviewer Expertise:

NA

No decision status is available

# Reviewer report: Reviewer response for version 1

**DOI:** 10.21956/mep.19686.r29416

**Published:** 2018-10-06

**Authors:** Barbara Jennings

**Affiliations:** 1 Norwich Medical School

This review has been migrated. The reviewer awarded 4 stars out of 5 I agree with the previous reviewers, this is an interesting and well-written paper about an important topic. It will be of great interest to anyone engaged in the preparation, delivery, and quality assurance of written assessments. The introduction, discussion, and cited papers provide lots of useful background information about the principles of using single-best-answer items to examine formatively and summatively. The cross-over study design with randomisation are explained in detail. But given that conclusions are being drawn about whether item-writing-flaws have measurable impact, I would like to know about any study size calculations and effect-size predictions that were used in the experimental plan?

Reviewer Expertise:

NA

No decision status is available

# Reviewer report: Reviewer response for version 1

**DOI:** 10.21956/mep.19686.r29415

**Published:** 2018-10-03

**Authors:** Richard Hays

**Affiliations:** 1 James Cook University

This review has been migrated. The reviewer awarded 4 stars out of 5 I enjoyed reading this paper, which is very practical and grounded in the real world of managing assessment. The outcome - that MCQ item writing flaws are a varied bunch of small things that can have variable impact on the choice of correct answer - is not surprising. This is of course just one institution and a relatively small sample of MCQ items, and I suspect that the quality of items and experience of item writers would vary considerably. That is the norm, although I expect expect that item writing workshops have been conducted. It is some time since the book on MCQ item flaws was written and, while I think the information is still relevant, item banks have probably improved and more subtle flaws may now be more common. The good news is that there may be little or no impact on overall examination performance, but how sure can we be about that? Item flaws are a source of error, and even 1 mark can be significant right on the cut score. This is an assessment QA activity that should probably be done more often, although there are likely to be resource constraints. In the meantime, item writers need to continue to avoid building in this source of error.

Reviewer Expertise:

NA

No decision status is available

# Reviewer report: Reviewer response for version 1

**DOI:** 10.21956/mep.19686.r29414

**Published:** 2018-10-02

**Authors:** P Ravi Shankar

**Affiliations:** 1 American International Medical University

This review has been migrated. The reviewer awarded 4 stars out of 5 This is an interesting paper. Constructing good MCQs is important for assessment. The authors describe their methodology well though I must admit I found some of the statistics difficult to follow. Single-response MCQs are widely used for various assessments including high-stakes examinations. Common flaws while constructing MCQs were well described. I did not fully understand about eponymous terms and how these could impact the MCQ scores and would be grateful for a simple explanation. All medical educators and faculty members will find this article to be of interest.

Reviewer Expertise:

NA

No decision status is available
